# *Juncus quartinianus* (Juncaceae, sect. *Ozophyllum*): A Neglected Species from the Horn of Africa and Its Re-Description Based on Morphological SEM Studies

**DOI:** 10.1371/journal.pone.0167838

**Published:** 2017-01-09

**Authors:** Anna Faltyn, Anna Jakubska-Busse, Paweł Jarzembowski, Jarosław Proćków

**Affiliations:** 1 Department of Plant Biology, Institute of Biology, Faculty of Biology and Animal Science, Wrocław University of Environmental and Life Sciences, Wrocław, Poland; 2 Department of Botany, Institute of Environmental Biology, Faculty of Biological Sciences, University of Wrocław, Wrocław, Poland; National Cheng Kung University, TAIWAN

## Abstract

*Juncus quartinianus* (Juncaceae sect. *Ozophyllum*) was described by Richard in 1851 from Ethiopia. Some authors have treated this species as a synonym of *J*. *fontanesii* and others as a synonym of *J*. *oxycarpus*. Based on morphological analyses of flowers, fruit and seeds, we propose to restore *J*. *quartinianus* as a distinct species from both these taxa. Its detailed re-description and an identification key to the morphologically similar species of *Juncus* sect. *Ozophyllum* are provided.

## Introduction

The genus *Juncus* L. [1] is a group of widely distributed perennial or rarely annuals herbs, which is divided into two subgenera and ten sections. It contains approximately 315 species, with centres of diversity in temperate zones [2–4]. Section *Ozophyllum* Dumort. [5], the largest in the genus *Juncus*, consists of annuals or rhizomatous to caespitose perennials with terete stems and leaf blades that are perfectly or imperfectly septate. The section comprises about 84 species with centres of distribution in eastern North America, southwestern Europe and the Far East [2, 6].

*Juncus oxycarpus* was described by Kunth [7]. According to the latest available monograph of the Juncaceae family [2] and Juffe [8], *J*. *oxycarpus* occurs in Africa from Southern Sudan, Ethiopia, Eritrea and Cameroon to South Africa. Plants from Eritrea, Ethiopia, Kenya and even Tanzania used to be referred to in the literature as *Juncus fontanesii* J. Gay ex Laharpe [9]. Kirschner *et al*. [2] reported that some specimens from Ethiopia and Eritrea that are classified as *J*. *oxycarpus* have longer capsules and are similar to *J*. *fontanesii* subsp. *pyramidatus* (Laharpe) Snogerup, which occurs in the eastern Mediterranean eastwards to Iraq, in North Africa from Tunisia eastwards, and in the Arabian Peninsula. Indeed, in the course of studying the herbarium material of *Juncus oxycarpus* and *J*. *fontanesii* subsp. *pyramidatus*, some unusual specimens from Ethiopia, Eritrea and Somalia were found. These were previously identified mainly as *J*. *oxycarpus*, but differ in their capsules that are longer than the perianth and their quite different seed coat surface. Some specimens were initially classified as *J*. *fontanesii*; however, we found that they do not belong to the latter species, because *J*. *fontanesii* possesses anthers that are distinctly longer than filaments but in the studied plants from Ethiopia, Eritrea and Somalia the situation is reversed (the anthers are shorter than the filaments or rarely, the anthers and filaments are equally sized).

Thus, our detailed morphological studies show that these specimens represent a distinct species. After careful analysis of the literature and herbarium material, it was found that this species was named *Juncus quartinianus* A. Rich. [10]. Type specimens were collected by R. Quartin-Dillon and A. Petit in Chiré (Ethiopia) in July 1844. This taxon was distinguished according to the following features: sessile, lateral single head, lanceolate, acute, subcastaneous tepals, and the inner ones slightly longer than the outer, six stamens one-third as long as the tepals and pyramidal capsules longer than the perianth [10]. According to Buchenau [11], type specimens have erect, slender and delicate stems, two flower heads with 6–16 flowers, pale green tepals, the inner conspicuously longer than the outer, capsules about one-third longer than the perianth and seeds that are similar to those of *J*. *articulatus* L. The same author in 1890 mentioned that specimens collected by Schimper represent a form of *Juncus fontanesii* with greenish flowers, and the material collected by Quartin-Dillon and A. Petit is similar to both *J*. *fontanesii* and *J*. *articulatus* [12]. Baker [13] placed *J*. *quartinianus* as a synonym of *J*. *fontanesii*, which according to the author, occurred in Eritrea, Ethiopia, British East Africa (currently Kenya and Uganda), German East Africa (a part of present Tanzania), British Central Africa (present Malawi), in the Mediterranean region and western Asia. Four years later, Buchenau [14] suggested that specimens classified as *J*. *quartinianus* probably represent *J*. *oxycarpus*, but some specimens from Ethiopia, Eritrea and even Kenya were still classified as *J*. *fontanesii*. Subsequent research, however, shows that this latter taxon is restricted only to the Mediterranean region and in fact, does not extend to Ethiopia and Eritrea [15]. Therefore, according to Carter [16], specimens from the type collection of *J*. *quartinianus* differ from specimens of *J*. *oxycarpus* only by having pale-green tepals and brown fruits, and thus, “these two names must be regarded as synonyms”.

The main goal of the present paper is to show that *J*. *quartinianus* is a well-defined species that occurs in Ethiopia, Eritrea and Somalia. Another purpose of our article is to indicate the differences among three taxa: *J*. *quartinianus*, *J*. *oxycarpus* and *J*. *fontanesii* subsp. *pyramidatus*.

## Materials and Methods

In total, 56 specimens (from 18 herbarium sheets) of *J*. *quartinianus*, 487 specimens (from 259 herbarium sheets) of *J*. *oxycarpus* (S1 Appendix) and 260 (from 86 herbarium sheets) of *J*. *fontanesii* subsp. *pyramidatus* (S2 Appendix), from the following 21 herbaria: B, BEI, BR, E, ETH, FR, H, JE, K, L, LG, LISU, MO, POZG, PRE, S, SAM, UPS, WAG, WRSL and WU were revised. For statistical analysis, we selected a representative subset of specimens from the geographic range of these three taxa. They are marked with an asterisk (*) after the herbarium acronym in the citations of representative specimens.

### Statistical analyses

The statistical analyses are based on 38 specimens of *J*. *quartinianus*, 71 specimens of *J*. *oxycarpus* and 75 specimens of *J*. *fontanesii* subsp. *pyramidatus*. Measurements were conducted only on mature specimens using a Nikon SMZ 800 stereoscopic microscope. We analysed 19 qualitative and quantitative characters, including two describing ratios between characters (Table 1).

**Table 1:** A list of morphological characters recorded.

Abbreviation	Character
Quantitative characters	
HP	Plant height (cm)
IL	Length of inflorescence (cm)
OTL	Length of outer tepal (mm)
OTMW	Width of outer tepal scarious margin (mm)
ITL	Length of inner tepal (mm)
ITSM	Width of inner tepal including scarious margin (mm)
ITEM	Width of inner tepal without scarious margin (mm)
ITMW	Width of inner tepal scarious margin (mm)
AL	Length of anther (mm)
FL	Length of filament (mm)
AL/FL	Ratio between length of anther and length of filament
CL	Length of capsule (mm)
CML	Length of capsule mucro (mm)
CW	Width of capsule (mm)
CL/TP	Ratio between length of capsule and length of perianth
NHI	Number of heads in the inflorescence
NFH	Number of flowers in the head
NF	Number of filaments
Qualitative characters	
SC	Shape of capsule

The width of the structure was measured at the widest point and the length of capsule and tepals from the base to the top. The inflorescence was measured from the base to its top. Plant height was measured from the base of the culm to the top of the inflorescence. When a character was present more than once per individual (e.g., tepal length), we measured that with the greatest value [3, 6]. Each specimen was treated as an Operational Taxonomic Unit (OTU). Means and standard deviation for 17 quantitative characters were calculated. One-way analysis of variance (ANOVA) followed by Tukey’s honestly significant difference (HSD) post hoc test for unequal sample sizes were used. Next, principal component analysis (PCA) based on the correlation matrix was performed for all quantitative characters. The analysis was used to examine the general variation as well as to reduce the set of characters that were correlated the strongest with the principal components. The missing data were replaced by means. Components in this analysis were extracted by a scree test. We selected the characters with the highest factor loadings in the first three components (r > 0.60). Prior to PCA, the measurements were standardised. Statistical analyses were performed using Statistica version 12 [17].

### Seed micromorphology

Seed morphology and ultrastructure were studied using scanning electron microscopy (SEM) in the Laboratory of Microscopic Techniques of the Faculty of Biological Sciences, University of Wrocław, Poland. A total of 33 seeds (12 of *J*. *quartinianus*, six of *J*. *oxycarpus* and 15 of *J*. *fontanesii* subsp. *pyramidatus*) was examined. Seeds were mounted to the microscope stub by double-adhesive tape; coated with carbon and silver particles; and examined using a Tesla BS-300 instrument. Each seed was measured using tpsDig version 2.0 software [18]. The material used for SEM imagery was collected from the selected herbarium specimens from E, ETH, FR and K (S1 Table).

## Results

### Statistical analyses

The results of the one-way ANOVA showed significant differences among 16 examined characters (Table 2). The characters accounting for the most morphologic dissimilarity were the ratio between length of capsule and length of perianth; length of filament; ratio between length of anther and length of filament; length of capsule and capsule mucro (Table 2). The results of Tukey’s HSD test are presented in supplemental material (S2 Table).

**Table 2:** Means ± standard deviation of various characters of *Juncus oxycarpus*, *J*. quartinianus and *J*. *fontanesii* subsp. *pyramidatus* and results of one-way ANOVA (high value of F are given in bold). For abbreviations, see Table 1.

Character	*J*. *oxycarpus*	*J*. *quartinianus*	*J*. *fontanesii* subsp. *pyramidatus*	ANOVA F value	p value
HP	44.45±16.2	17.69±6.42	25.31±9.61	36.954	<0.05
IL	7.4±3.58	3.55±1.67	4.33±2.14	23.989	<0.05
OTL	3.51±0.53	3.38±0.27	3.2±0.36	9.659	<0.05
OTMW	0.3±0.07	0.3±0.08	0.2±0.05	22.440	<0.05
ITL	3.42±0.56	3.45±0.29	3.13±0.33	10.353	<0.05
ITSM	0.92±0.16	1.02±0.12	0.95±0.08	5.852	<0.05
ITEM	0.53±0.08	0.63±0.07	0.56±0.07	14.161	<0.05
ITMW	0.25±0.07	0.25±0.05	0.24±0.05	0.570	>0.05
AL	0.63±0.12	0.74±0.12	0.97±0.21	73.398	<0.05
FL	1.07±0.15	0.87±0.14	0.55±0.12	**239.311**	<0.05
AL/FL	0.6±0.13	0.87±0.17	1.84±0.66	**152.749**	<0.05
CL	2.97±0.61	4.05±0.27	4.21±0.46	**114.024**	<0.05
CML	0.34±0.11	0.53±0.1	0.78±0.2	**140.678**	<0.05
CW	1.38±0.23	1.67±0.12	1.25±0.19	31.928	<0.05
CL/TP	0.84±0.1	1.19±0.12	1.37±0.12	**268.997**	<0.05
NHI	7.9±5.98	3.13±1.59	11.23±8.47	15.746	<0.05
NFH	20.27±6.28	18.1±8.16	10.9±4.24	40.014	<0.05

PCA performed based on 18 quantitative characters yielded three components, which accounted for 68.9% of total variation. The first component accounted for 36.16% of total variation, while the second represented 21.86% of total variation and the third 10.92% of total variation. Eleven analysed characters had high positive factor loadings (> 0.60) and five high negative loadings (< -0.60). The characters, which showed the greatest influence in the first component, were plant height (HP); length of anther (AL); length of filament (FL); ratio between length of anther and length of filament (AL/FL); length of capsule (CL); length of capsule mucro (CML); ratio between length of capsule and length of perianth (CL/TP); number of flowers in head (NFH); and number of filaments (NF). Length of outer and inner tepals (OTL and ITL); width of inner tepal (including scarious margin (ITSM)); width of inner tepal without scarious margin (ITEM); and width of capsule (CW) showed the greatest influence in the second component. Length of inflorescence (IL) and number of heads in the inflorescence (NHI) were highly correlated with the third component (Table 3).

**Table 3:** Results of the principal component analysis for *Juncus oxycarpus*, *J*. quartinianus and *J*. *fontanesii* subsp. *pyramidatus* (values with the highest factor loadings (> 0.60) are given in bold). For abbreviations, see Table 1.

Character	Component 1	Component 2	Component 3
HP	**-0.6547**	-0.2203	-0.5221
IL	-0.5196	-0.1781	**-0.7041**
OTL	-0.3073	**0.6566**	-0.3521
OTMW	-0.5107	0.4552	0.0734
ITL	-0.2531	**0.7916**	-0.2515
ITSM	0.1968	**0.8162**	-0.0691
ITEM	0.2340	**0.7281**	0.0009
ITMW	-0.0452	0.5835	-0.2130
AL	**0.7173**	0.0639	-0.3350
FL	**-0.8689**	0.0734	0.0984
AL/FL	**0.8238**	-0.0676	-0.4073
CL	**0.7935**	0.4552	-0.0723
CML	**0.8083**	0.0603	-0.0839
CW	-0.1651	**0.7267**	0.1642
CL/TP	**0.9324**	0.0439	0.0599
NHI	0.1980	-0.3287	**-0.7858**
NFH	**-0.6185**	0.3991	-0.0652
NF	**0.8630**	0.1711	0.0404
Total variance (in %)	36.16	21.86	10.92

**Fig 1:**
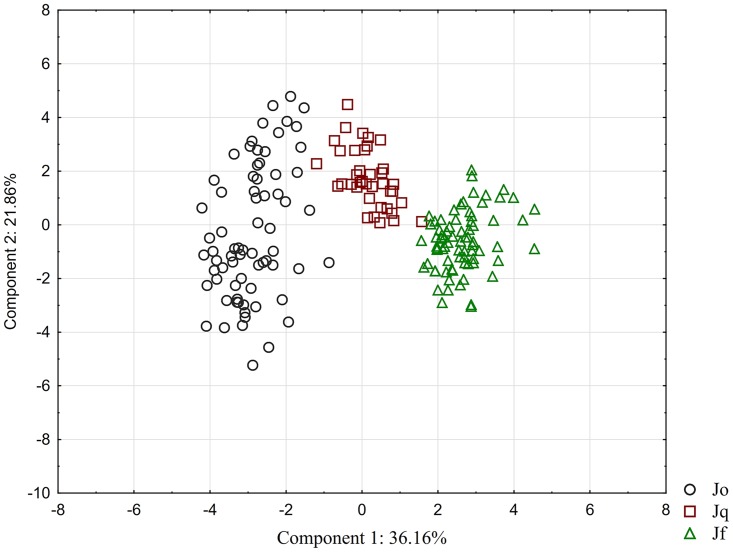
Scatter plot of the component scores of principal component analysis loadings I and II of 184 specimens. Abbreviations: Jo–*Juncus oxycarpus*, Jq–*J*. *quartinianus*, Jf–*J*. *fontanesii* subsp. *pyramidatus*.

The scatter plot according to the first two factors revealed three neighbouring or slightly overlapping groups of specimens, which corresponded to the three taxa (Fig 1).

### Seed micromorphology

**Fig 2:**
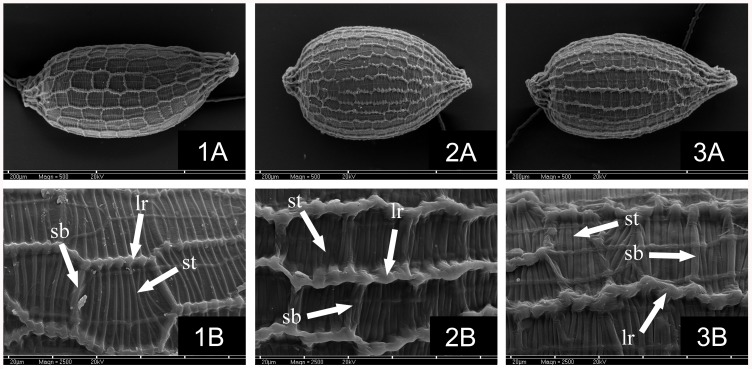
Scanning electron microscopy (SEM) photographs of seeds. (A) Entire seed. (B) Seed-surface details. (1) *Juncus oxycarpus*. (2) *J*. *quartinianus*. (3) *J*. *fontanesii* subsp. *pyramidatus*. (Lr) longitudinal ridges, (st) short transverse ridges, (sb) short border transverse ridges.

The seeds of the three taxa are similar in the size, but they have different seed coat surfaces. The seeds of *J*. *oxycarpus* are longitudinally 14–20 striate (lr), with short transverse ridges (st) that do not adhere to each other, short border transverse ridges (sb) usually pronounced, and formed by single thicker ridges (about 2.0–3.5 times that of other transverse ridges (st)). The seed coat surface of *J*. *fontanesii* subsp. *pyramidatus* is longitudinally (18)20–24(26) striate (lr). However, their short transverse ridges (st) do not adhere or sometimes adhere to one another but, in many cases, not so tightly. Moreover, their short border transverse ridges (sb) (between longitudinal ridges) are usually faint and are formed by single, slightly thicker ridges. The seed coat surface of *J*. *quartinianus* is distinctly longitudinally (18)20–24(26) striate (lr), with short transverse ridges (st) (between longitudinal ridges), usually adhering to one another (i.e., they are very tightly arranged). The short border transverse ridges (sb) are usually pronounced (rarely faint) and formed by 2**–**3 adherent ridges of the same thickness like other transverse ridges (st) or are thinner (Fig 2).

## Discussion

The analysis results confirmed that *J*. *quartinianus*, *J*. *oxycarpus* and *J*. *fontanesii* subsp. *pyramidatus* are differentiated. In the past, this first taxon was treated originally as a synonym of *J*. *fontanesii* [13] and later as a synonym of *J*. *oxycarpus* [16].

**Fig 3:**
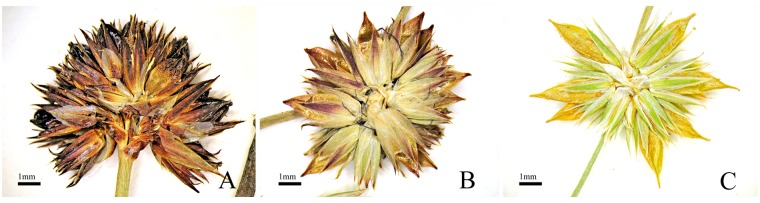
Flower heads with capsules. (A) *Juncus oxycarpus*, (B) *J*. *quartinianus*, (C) *Juncus fontanesii* subsp. *pyramidatus*.

We found in the herbarium material four sheets initially identified as *J*. *fontanesii* and then as *J*. *oxycarpus*, and the rest of the specimens were wrongly classified as this latter species. Our research indicated the diagnostic value of the characters, which distinguished these three taxa. One of the significant features providing differentiation between *J*. *quartinianus* and *J*. *oxycarpus* is ratio of the capsule and perianth length. *Juncus quartinianus* has capsules longer than the perianth or, extremely rarely in some flowers, only slightly longer than the perianth. *Juncus oxycarpus* capsules are usually equally long or shorter than the perianth or, rarely, are slightly longer than the perianth (Fig 3).

*Juncus fontanesii* subsp. *pyramidatus* has capsules longer than the perianth, but *J*. *quartinianus* can be distinguished easily from the latter taxon by oblong-ovoid, apiculate or rarely ovoid and acute capsules as well as anthers that are shorter than the filaments (or, exceptionally, equal in size or slightly longer than the filaments), whereas *J*. *fontanesii* subsp. *pyramidatus* produces capsules that are narrowly pyramidal to subprismatic, gradually narrowing into the rostrum, and, generally, the anthers are distinctly longer than the filaments. The main differences among the three taxa are summarised in Table 4.

**Table 4:** Comparison of the main diagnostic characters of *Juncus oxycarpus*, *J*. *quartinianus* and *J*. *fontanesii* subsp. *pyramidatus* (distinguishing characters are underlined).

Characters	*Juncus oxycarpus*	*Juncus quartinianus*	*Juncus fontanesii* subsp. *pyramidatus*
**Capsule length (mm)**	(1.9)2.1–3.95(4.15)	(3.25)3.5–4.25(4.45)	(3.05)3.35–5.0(5.25)
**Capsule/perianth length ratio**	(0.56)0.68–1.02(1.04)	(0.99)1.05–1.35(1.49)	1.18–1.54(1.69)
**Capsule shape**	oblong–ovoid to ovoid, subobtuse, apiculate, rarely acute	oblong–ovoid, apiculate, rarely ovoid and rarely acute	narrowly pyramidal to subprismatic, gradually narrowing into rostrum
**Capsule vs. perianth length**	capsule much shorter, equal or slightly longer than perianth	capsule longer than perianth or rarely, slightly longer than perianth (only in some flowers)	capsule conspicuously longer than perianth
**Number of stamens**	3, less often 4, 5, 6	6, less often 3, 4, 5	6
**Anther/filament length ratio**	0.29–0.94(1.1)	(0.48)0.59–1.18(1.23)	(0.9)1.03–2.66(2.71)
**Seed coat surface**	longitudinally 14–20 striate (lr)	longitudinally (18)20–24(26) striate (lr)	longitudinally (18)20–24(26) striate (lr)
	short transverse ridges (st) (between longitudinal ridges) always do not adhere to each other	short transverse ridges (st) (between longitudinal ridges) usually adhere to each another (i.e., very tightly arranged)	short transverse ridges (st) (between longitudinal ridges) do not adhere or sometimes adhere to each another (but in many cases not so tightly)
	short border transverse ridges (sb) (between longitudinal ridges) usually pronounced, and formed by single thicker ridges (about 2.0–3.5 times that of other transverse ridges (st))	short border transverse ridges (sb) (between longitudinal ridges) usually pronounced (rarely faint), formed by 2–3 adherent ridges of the same thickness like other transverse ridges (st) or thinner	short border transverse ridges (sb) (between longitudinal ridges) faint, usually formed by single slightly thicker ridges (up to about two-times that of other transverse ridges (st))

The most conspicuous difference among the taxa is the seed coat surface. The sculpture of the seeds is a distinct diagnostic character that is useful for distinguishing *Juncus* taxa [4, 19]. The *J*. *quartinianus* seed coat surface is distinctly longitudinally (18)20–24(26) striate, with short transverse ridges usually adhering to one another, and the seeds of *J*. *oxycarpus* are longitudinally 14–20 striate, with short transverse ridges that do not adhere to each other. Thus, the seeds of *J*. *quartinianus* more resemble the seeds of *J*. *fontanesii* subsp. *pyramidatus*, which also are longitudinally (18)20–24(26) striate. However, the short transverse ridges of the latter seeds do not adhere or sometimes adhere to one another but, in many cases, not so tightly. Moreover, their short border transverse ridges are usually faint and are formed by single slightly thicker ridges. The seeds of *J*. *quartinianus* have short transverse ridges that usually adhere to one another, and their short border transverse ridges are usually pronounced and formed by 2**–**3 adherent ridges of the same thickness, like other transverse ridges, or are thinner (Fig 2, Table 1).

Our detailed analysis shows that *J*. *quartinianus* should be recognised as a separate species; therefore, we propose to restore it.

Revision of the herbarium material revealed that Richard’s [10] description of *J*. *quartinianus* is inaccurate, since, according to the author, this species should have a lateral single flower head and inner tepals slightly longer than the outer ones. However, some specimens from the type material (and other plants that belong to the species) have 2–5(6) heads on the top of the stem, and the tepals are equal in size in some flowers. The other distinguishing characters are the capsule shape and the ratio of its length to the perianth. Richard [10] mentioned that capsules of *J*. *quartinianus* are pyramidal, apiculate, glabrous and longer than the perianth. Indeed, plants of this species have apiculate or rarely acute fruit that is longer than the perianth; however, they are oblong-ovoid (including type specimens) and are rarely ovoid. Another character added by Richard [10] in the diagnosis is the presence of six stamens, which are 3 times shorter than the tepals, but in the description below the diagnosis, he stated that the stamens are 2 times shorter than the tepals. We found that this is a more correct feature, including for type specimens, which have a ratio of stamen to tepal length of about 1:2 (nevertheless, the proportion of 1:3 is also possible in the species). Moreover, according to Richard’s diagnosis [10], perianth segments should be lanceolate, acute, subcastaneous and the inner tepals slightly longer than the outer ones. However, our study revealed that in flowers of the species (including the type specimens), there are six (or, less often, three), four or five stamens; the tepals are broadly lanceolate to ovate-lanceolate, acuminate to acute, equal or the inner longer, and all tepals are greenish-red or reddish to dark red or greenish, usually with a red apex and often with additional red stripes along the margins.

### Taxonomy

*Juncus quartinianus* Rich. (distinguishing characters underlined)

Type ETHIOPIA. Abyssinie, Ediofia, Chiré [Shire], 1844, *R*. *Quartin-Dillon & A*. *Petit*, (holo-: P 450405 (available online)!; iso-: K 0345815!, K000345816!, L, P 450406 (available online)!, P 450407(available online)!, WAG 1717!)

The numbers in parentheses are the minimum and maximum values measured for the individual characters.

**Fig 4:**
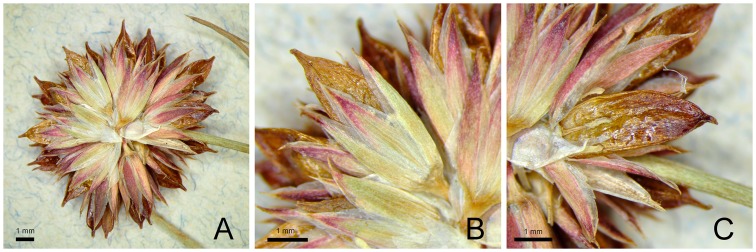
*Juncus quartinianus* (S 08–19215). (A) Flower head. (B) Flower with ripe fruit. (C) Capsule and stamens. Photo *P*. *Jarzembowski*.

Perennial herbs, 5–21(40) cm tall, sometimes with above-ground stolons, cataphylls zero to two. Cauline leaves two to five, terete, unitubular, distinctly perfectly septate, basal leaves sometimes reaching the inflorescence. Auricles obtuse, of variable length (0.5)0.8–2.35(2.5) mm, yellowish-subcoriaceous to scarious. Inflorescence 1.0–5.7(8.0) cm long, of 1–5(6), (3)5–27(43)-flowered heads. Lower bract leaf-like, 1.0–4.6 mm long, shorter, less often longer, or rare subequalling inflorescence. Tepals usually equal or inner sometimes slightly longer than outer (in some flowers), broadly lanceolate to ovate–lanceolate, inner (2.55)2.85–3.85(4.2) mm long, outer (2.6)2.75–3.75(3.9) mm long, acute to acuminate, sometimes slightly recurved at apex, these are usually greenish-red or reddish to dark red or greenish and with usually a red apex and often with additional red stripes along the margins; margins membranous 0.17–0.41(0.55) mm wide. Stamens six (less often three, four or five), usually about two-times (less often until three-times) shorter than the tepals, anthers (0.5)0.58–(0.9)0.96 mm long, shorter than (0.52)0.64–1.14(1.2) mm filaments, rarely, the anthers are slightly longer than the filaments and very rarely, the anthers and filaments are equal in length (anthers/filaments length ratio: (0.48)0.59–1.18(1.23)). Capsule oblong–ovoid, apiculate, rarely ovoid and acute, brown to golden-brown, shining, trigonous, unilocular, (3.25)3.5–4.25(4.45)×(1.37)1.46–1.87(1.92) mm with rostrum (0.3)0.4–0.7(0.82) mm long, longer than perianth or rarely only slightly longer than perianth (seldom in some flowers) (Fig 4A–4C). Seeds ellipsoid–ovoid, 0.35–0.51×0.22**–**0.29 mm, pale brown, longitudinally (18)20–24(26) striate (lr), short transverse ridges (st) (between longitudinal ridges) usually adhere to one another (i.e., very tightly arranged), and short border transverse ridges (sb) (between longitudinal ridges) usually pronounced (rarely faint), formed by two to three adherent ridges of the same thickness like other transverse ridges or thinner; appendages absent (Fig 2A–2B).

Representative specimens examined (*specimens measured for statistical analyses).

ETHIOPIA. Amhara Region: ca. 115 km North of Lalibela along the road to Sekota, 12°33’N 39°04’E, 2,050 m, 27 Oct. 2001, *I*. *Fries*, *S*. *Bidgood*, *M*. *Wonderfrask & E*. *Getachew 10*,*804* (K*); Tigray Region: Adigerat (Adigrate) [Adigrat], 130 km S.S.E. of Asmara, 20 Jan. 1963, *T*.*H*. *Hages 170* (ETH*, K*); South of Sinikala (Ferawen), 2,500 m, 17 Apr. 1992, *S*. *Demissew 3191* (ETH*); ERITREA. Afdeyo, 2 km along road to the right, from turnoff 33 km N of Asmara along the road to Keren, 2,400 m, 29 Nov. 1989, *O*. *Ryding 2059* (ETH, UPS*); Asmera [Asmara] town, main campus area of Asmera University, 2,350, 4 Jun. 1989, *O*. *Ryding 1976* (ETH*, UPS*); Hamasien, 22 Jan. 1916, *J*. *Galdrat s*.*n*. (K*); Saganeïti, Gorge de Degerra, 2,200 m, 10 Mar. 1892, *G*. *Schweinfurth & D*. *Riva 892* (BR*, K*, P (available online), S*); SOMALIA.‘Somaliland’, Dadan, 48°38’ E 10°57’N, 5,000’, 13 Aug. 1957, *J*.*G*.*B*. *Newbould 912* (K*); Sanaag: escarpment S of Laasqoray near Moon, 11:01N 48:25E, 1,300 m, 16 Jan. 1995, *M*. *Thulin*, *A*. *Dahir & A*. *Hassan 9072* (K*, UPS*).

### Distribution and habitat

Eritrea, Ethiopia and Somalia, in wet places on sandy soil, limestone along streams, bogs near water holes, clay soil, seasonally seepage ground, the margins of small ponds, dry river banks, at 1,300–2,500 m a.s.l.

### Phenology

The capsules usually mature from October to June. However, ripe fruits were also present on 13 August 1957 (*Newbould 912*, plants from Somalia).

The identification key to *Juncus quartinianus* and other morphologically similar species of *Juncus* sect. *Ozophyllum* (on the basis of Kirschner *et al*. [2]). The characters that are new in the key are underlined).

All the species belong to the group with “leaves not polygonal nor sulcate in transverse section, at most weakly many-striate” [i.e., the feature No. ‘19:’ in a key to sect. *Ozophyllum* in Kirschner *et al*. p. 153[2]).

1. Stamens usually 3, sometimes in some flowers to 6; capsule equalling, shorter (or rarely slightly longer than perianth) ………………………………………..…………..………***J*. *oxycarpus***

- Stamens usually 6; capsule exceeding to greatly exceeding perianth……………..….……..2

2. Anthers shorter than or rarely equalling filaments..…………………………….……………6

- Anthers usually 1.2 times or more longer than filaments …..………………….…***J*. *fontanesii***

3. Plants caespitose or with long above-ground stolons, not mat-forming; rhizomes absent or short, sparsely branched, not ascending; ………………………………………………….……4

- Plants mat-forming, with short branched ascending rhizomes; …….…. **subsp. *brachyanthus***

4. Rhizome present, short; stolons short or absent; capsule slightly exceeding perianth, narrowly pyramidal to trigonous–ovoid, abruptly or ± gradually contracted into a mucro 0.4–1.1 (1.6) mm long…………………………………………………………………………..…5

- Rhizome absent; stolons very long, to 2 m; capsule distinctly exceeding perianth, trigonous–ovoid, tapering into a mucro 1–2 mm long……………………………………………….**subsp. *fontanesii***

5. Plants usually 15–40 cm tall; tepals usually lanceolate (less often broadly lanceolate), with narrow scarious margins; capsule with a mucro 0.4**–**1.1(1.6) mm long……………………………………………………..……………….…**subsp. *pyramidatus***

- Plants usually 8–15 (rarely to 25) cm tall; tepals ovate, with broad scarious margins; capsule with a mucro 0.4–0.8 mm long ….………………………………………………**subsp. *kotschyi***

6. Inflorescence with 1–6 flower heads; all tepals acute to acuminate, inner sometimes slightly longer than outer ones; capsule trigonous, oblong–ovoid, apiculate or rarely ovoid and acute, tapering into a (0.3)0.4–0.7(0.82) mm-long rostrum; anthers shorter than filaments (exceptionally anthers slightly longer than filaments or anthers and filaments are equal) [Eritrea, Ethiopia and Somalia]…………………………….…………………….***J*. *quartinianus***

- Inflorescence with (1)10–35(80) flower heads; outer tepals acute or rarely subobtuse and mucronate, sometimes slightly longer than inner ones; inner tepals obtuse to acute, often mucronate; capsule trigonous–ovoid, distally acute to subobtuse, subabruptly, tapering into a 0.3 mm-long rostrum; anthers equalling filaments to slightly longer than filaments ………………………………………………………..………***J*. *articulatus* subsp. *articulatus***

## Supporting Information

S1 Appendix Specimens of *Juncus oxycarpus* examined. (DOCX)

S2 Appendix Specimens of *Juncus fontanesii* subsp. *pyramidatus* examined. (DOCX)

S1 Table Specimens used in the SEM examination of seeds. (DOCX)

S2 Table Results of Tukey’s HSD test for unequal sample sizes. (DOCX)

## References

[1] Linnaeus C. Species plantarum exhibentes plantas rite cognitas, ad genera relatas, cum differentiis specificis, nominibus trivialibus, synonymis selectis, locis natalibus, secundum systema sexuale digestas. Holmiae Impensis Laurentii Salvii; 1753.

[2] Kirschner J, compiler. Juncaceae 2: Juncus subg. Juncus, Species Plantarum: Flora of the World. Part 7. Canberra; 2002.

[3] Knapp WM, Naczi RFC. Taxonomy, morphology, and geographic distribution of Juncus longii (Juncaceae). Syst Bot. 2008;33: 685–694.

[4] Abdel Khalik KN. Seed coat morphology and its systematic significance in *Juncus* L. (Juncaceae) in Egypt. J Syst Evol. 2010;48(3): 215–223.

[5] Dumortier BC. Florula Belgica operis maioris Prodromus. Tornaci Nerviorum: Casterman; 1827.

[6] Knapp WM. *Juncus fascinatus* (Juncaceae), a new combination in *Juncus* sect. *Ozophyllum* and notes on morphologically similar species. Phytotaxa. 2014;174(5): 243–260.

[7] Kunth CS. Enumeratio plantarum omnium hucusque cognitarum: secundum familias naturales disposita, adjectis characteribus, differentiis et synonymis. Vol. 3, Stutgardiae et Tubingae [Stuttgart, Tübingen]: Sumptibus J.G. Cottae; 1841.

[8] Juffe D. Juncus oxycarpus. The IUCN Red List of Threatened Species 2015: e.T185311A85695891. 2015. Available: http://www.iucnredlist.org/details/185311/0

[9] De Laharpe J. Essai d’une Mongraphie des Vrais Joncées, Comprenant les Genres Juncus, Luzula et Abama. Mém Soc Hist Nat Paris. 1825;3: 89–179.

[10] Richard A. Tentamen florae Abyssinicae: seu, Enumeratio plantarum hucusque in plerisque Abyssiniae provinciis detectarum et praecipue a beatis doctoribus Richard Quartin Dillon et Antonio Petit (annis 1838–1843) lectarum, Vol. 2 Parisiis: Arthus Bertrand; 1851.

[11] Buchenau F. Die Verbreitung der Juncaceen über die Erde In: Engler A, editor. Botanische Jahrbücher für Systematik, Pflanzengeschichte und Pflanzengeographie. Leipzig: Verlag von Wilhelm Engelmann; 1880;1: 104–141.

[12] Buchenau F. Monographia Juncacearum In: Engler A, editor. Botanische Jahrbücher für Systematik, Pflanzengeschichte und Pflanzengeographie. Leipzig: Verlag von Wilhelm Engelmann; 1890;12:1–495.

[13] Baker JG. Juncaceae In: Thiselton-Dyer WT, editor. Flora of Tropical Africa: Pontederiaceae to Cyperaceae Vol. 8 The Oast House, Brook, Ashford, Kent; Lovell Reeve & Co.; 1902 p. 91–97.

[14] Buchenau F. Juncaceae In: Engler A, editor. Das Pflanzenreich IV. 36 Leipzig: Verlag von Wilhelm Engelmann; 1906 pp. 1–248.

[15] Weimarck H. Studies in Juncaceae with special reference to the species in Ethiopia and the Cape. Svensk Bot Tidskr. 1946;40(2):141–178.

[16] Carter S. Notes on Tropical African Juncaceae. Kew Bull. 1965;19(3): 513–515.

[17] StatSoft, Inc. STATISTICA (data analysis software system). Version 12. 2013.

[18] Rohlf FJ. TpsDig (Digitize coordinates of landmarks and capture outlines). Version 2.16. Stony Brook, Ecology and Evolution, SUNY. 2010. http://life.bio.sunysb.edu/morph/morphmet.html.

[19] Kirschner J, Rejdali M, Drábková L. A new *Juncus* of the section *Tenageia* from Morocco and Egypt. Preslia 2004;76: 371–376.

