# Supplementary material for: Juncus quartinianus (Juncaceae, sect. Ozophyllum): A Neglected Species from the Horn of Africa and Its Re-Description Based on Morphological SEM Studies
Source: PLoS One. 2017 Jan 9;12(1):e0167838. doi: 10.1371/journal.pone.0167838 (PMC5221796; doi:10.1371/journal.pone.0167838)
Supplement: S1 Appendix — (DOCX) [file pone.0167838.s001.docx]

**S1 Appendix. Specimens of *Juncus oxycarpus* examined** (*****specimens measured for statistical analyses).

Type: [Cape Provinces] Liesbeek R., s.d., *C.H. Bergius* (syn-: WRSL); Paarl, Berg Rivier, s.d., *J.F. Drège a* (syn-: E 193985, K 307595**,** K 307596***,** L 795755, MO 2139114, P 450408 (available online), P 450409 (available online)).

*Juncus gentilis* N.E. Br., Kew Bull. 2: 83 (1914). T: Transvaal, Modderfontein, s.d., *P. Conrath 1173* (holo-: K 345822***)**.

*Juncus suboxycarpus* Adamson, J. Linn. Soc., Bot. 50: 14 (1935). T: Natal, Clairmont, s.d., *R. Schlechter 3043* (iso-: FR 28031, FR 28032, K 345823, PRE 91168-0, PRE 592298-0, WU 43487; para-: Pieter-Mauritz-burg [Pietermaritzburg], Drakenberg, Oct. 1841, *J. Wahlberg s*.*n*. (S)).

*Juncus oxycarpus* subsp. *sparganioides* Weim., Svensk Bot. Tidskr. 40: 166 (1946). T: Mt. Kenya, Liki, 11 Feb. 1922, *R.E. Fries & T.C.E. Fries 1477* (holo-: UPS**)**.

Representative specimens examined

ANGOLA. distr. do Huambo, nova Lisboa, arredores de Nova Lisboa, Caála (Belém), ca. de 1,700 m s m, 1 Sep. 1970, *M. de Silva 3250* (BR*); Serra de Chela, Humpata, 1,800 m, 25 Sep. 1941, *coll*. *unknown 13525* (B); BOTSWANA. Aedume Park, Gaborone Dam, 24º42’S 25º54’E, 1,050 m, 20 Oct. 1977, *O.J. Hansen 3246* (UPS); BURUNDI. Luvironza Urundi INEAC, 1,850 m, 12 Mar. 1958, *G. Michel 5204* (MO); Mwaro, Kisozi [Gisozi], 3°34’S 29°41’E, 2,200 m, 11 Apr. 1971, *J. Lewalle 5489* (BR); Muramwya, Ijenda, 2,000 m, 28 Dec. 1971, *M. Reekmans 1304* (LG, MO**)**; Muramvya, entre Ijenda et Mwaro, 25 Oct. 1970, *J. Lewalle 4876* (BR); Muramvya, Mugongo, crête au N du mont Maga, 2,200 m, 4 Jun. 1966, *J. Lewalle 885* (BR); Muramwya, Ryarusera (mission), 2,100 m, 27 Feb. 1972, *M. Reekmans 1576* (LG); Muyange, 2,000 m, 12 Sep. 1971, *M. Reekmans 1037* (LG*, MO***)**; Ngozi, Campazi, Mont Mikiko, 2°59’S 29°32’E, 2,450 m, 18 Apr. 1979, *M. Reekmans 7846* (LG***)**; Ngozi, Rwegura, 2°55’S 29°33’E, 2,000 m, 28 Sep. 1976, *M. Reekmans 5360* (LG); CAMEROON. N. Région: E, Kumbo, 1,600 m, 1 Feb. 1970, *Staff National d’Agriculture de Dschang* (K); DEMOCRATIC REPUBLIC OF THE CONGO. Cratère du Biviro, 2,400 m, Oct. 1937, *J. Vebrun 7906* (K); 2 km de Luoni[l]e (Plateau des Marungu, Katanga), 16 Feb. 1970, *S. Lisowski, F. Malaisse & J.-J. Symoens 9812* (BR); Mare Kasozia, en. de Kasiki (Plateau des Marungu, Katanga), 2,100 m, 10 Nov. 1970, *S. Lisowski, F. Malaisse & J.-J. Symoens 12105* (BR, POZ) & *12137a* (POZ); 3 km à l’west de Kasiki (Plateau des Marungu, Katanga), env. 2,000 m, 12 Jun. 1969, *S. Lisowski, F. Malaisse & J.-J. Symoens* *6148* (BR) & *6150* (BR, POZ**)**; Moushumangabo, 2,075 m, Aug. 1937, *J. Vebrun 7119* (B, K); Plaines de Laves Entre Les Lacs Kivu et Edouard, marais du cratère Tchikéré à la base S. du Mikeno, 2,200 m, Apr.-May 1929, *H. Humbert 8101* (BR*); ETHIOPIA: Abyssinie; bords du lac Zonai, Jul. 1909, *D. Rouss[..]an s.n.* (L***)**; Lagadodie River, 2,500 m, 10 May [19]87, *C. Parker 4503* (ETH**)**; Addis Ababa, hill behind British Embassy, 8,500 ft, 29 Nov. 1953, *H.F. Mooney 5058* (K***)**; Oromia Region. About 30 km NE. of Addis Ababa, along Asmara road, ±2,200m, 6 May 1966, *W.J.J.O. de Wilde & B.E.E. de Wilde-Duyfjes 10926* (WAG**)**; Arsi Zone: Chilalo awraja, 3 km S. of Asella at Livestock farm, ca. 2,400 m, 7 Oct. 1970, *M*. *Tulin 1390* (ETH, K**,** UPS); at base of Mt. Cilialo [Mount Chilalo] near Asella, ±2,000 m, 10 Sep. 1965, *W.J.J.O. de Wilde & B.E.E. de Wilde-Duyfjes 8048* (ETH**,** K**,** MO***,** WAG); 35 km. ESE of Shashamane, along the road to Dobollo, 7 03N 38 49 E, ±2,600 m, 26 Jul. 1970, *J.J.F.E. de Wilde 6849* (MO***,** WAG***)**; 32 km on the Shashemane-Dodola road, ca. 7º02’N 38º52’E, ca. 2,700 m, 15 May 1980, *M. Thulin, A. Hunde & M. Tadesse 3663* (UPS**)**; Bale Zone: Bale Mountains National Park, Dinsh [Dinsho], ca. 400 km S.E. of A.A., along river Wbb (oeb), 3,070 m, 16 Nov. 1979, *T. Mesfin 653* (ETH**)**; Dello Awraja, in Harenna Forest, Kecha, ca*.* 45 km on Dello Mena–Goba road*,* 6º42.5’N 39º44’E, 2,400 m, 10 Aug. 1986, *T. Mesfin 5058* (ETH**)**; 10 miles west of Dinchu (Curie), on Shashamane road, 3948:0706, 3,100 m, 19 May 1975, *J*. *Ash 2923* (K**)**; Gurie, 7º06’N 39º47’E, 10,600 ft, 23 Apr. 1958, *H.F. Money 7270* (ETH**,** K**)**; Gurie, 10,400 ft, 23 Apr. 1958, *H.F. Money 7268* (ETH***)**; ca. 40 km W of Robe on the main road, W of road junction to Bale Mt. Nat. Park Headquarters in Dinshu, 2,400 m, 7 Nov. 1982, *I*. *Kukkonen 12580* (H); East Shewa Zone: Mt. Zuquala (Zukwala) about 60 km S of Addis Ababa, ±2,900 m, 30 Oct. 1965, *W.J.J.O. de Wilde & B.E.E. de Wilde-Duyfjes 8556* (ETH**,** MO, WAG***)**; Mt. Zuquala (Zukwala), 74 km of SSE of Addis Ababa, inside the rim of crater, 8º32’N 38º51’E, 2,840–3,000 m, 1994, *Kristoffer & E*. *Hylander, A*. *Gobena 85* (ETH***,** UPS***)**; North Shewa Zone: about 5 km N. of Addis Abeba, ±2,400 m, 26 Mar. 1965, *W.J.J.O. de Wilde & B.E.E. de Wilde-Duyfjes 5948* (ETH**,** K**)**; about 100 km N. of Addis Ababa, between Fitche and Debra Libanos, ±2,000 m, 25 Apr. 1966, *W.J.J.O. de Wilde & B.E.E. de Wilde-Duyfjes 10837* (WAG); 145 km from Addis Ababa on the road to Dessie, 9º35’N 39º45’E, 20 Apr. 1964, *Lemma G. Selassie 142* (ETH***)**: Entoto Hill, just N. of Addis Ababa, 9º05’N 38º48’E, 2,700 m a.s.l., 14 Nov. 1972, *I. Friis 1174* (ETH***)**; about 5 km N of Addis Ababa, at base of Mt. Entoto, ±2,600 m, 31 Dec. 1965, *W. de Wilde 9529* (WAG***)**; Southern Nations, Nationalities, and Peoples’ Region. Sidama Zone: 10 km S. of Agere Selam [Hagere Selam], along the road to Kebre Mengist, 6º27’N 38º33’E, ±2,700 m, 20 Jul. 1970, *J.J.F.E. De Wilde 6705* (MO***,** WAG); About 10 km SE of Hagere Selam, SE of Wondo, ±3,000 m, 13 Mar. 1966, *W.J.J.O. de Wilde & B.E.E. de Wilde-Duyfjes 10312* (WAG); Lake Arakit, near Dilla village, on Hosanna road, square 32 B, 0752:3757, 2,850 m, 2 Nov. 1974, *J*. *Ash 2728* (K**,** MO**,** WAG); Wolayita Zone: near Wallome Sodo, 2,000 m, 22 Sep. 1969, *C. Parker E. s.n.* (K**)**; KENYA. Central Province. Pr. Forest Station (regio silvae montanae), 2,300 m, 26 Dec. 1921, *Rob E. och Th.C.E. Fries 331* (K, S***)**; Mt. Aberdare orient., 13 Mar. 1922, *Rob E. & Th.C.E. Fries 2278* (UPS***)**; Kiambu County: Kikuyu, Ondiri Swamp, 11 Apr. 1960, *Verdcourt 2649* (K); Kirinyaga County: Thiba R. crossing 2 m above fishing camp, 00°23’S 37°19’E, 1,980 m, 10 Nov. 1971, *S.A. Robertson 1622* (K, MO**)**; Nyeri County: Mt. Kenya, W slope, Naro Moro Track, 2,400-2,700 m, 2 Mar. 1972, *I. Backéus & L. Jonsson 323* (UPS***)**; Rift Valley Province. Bondui, Mau forest, 7,500’, 22 Jan. 1946, *P.R.O. Bally 4910* (K); Elgeyo-Marakwet County: Cherangani Hills, Forest below Kaisungor [Kaisungurr], 7,500 ft, 1 Oct. 1959, *Verdcourt 2435*(K***)**; Nakuru County: Karia Ndusi [Kariandus], near Lake Elmeteita [Lake Elmenteita], 6,000 ft, Dec., *G.F. Scott Elliot 6623* (K***)**; halway down Kedong Rift, 8 Dec. 1954, *B. Verdcourt 1161* (BR***,** S***)**; Turi area near Molo (Mr Start’s farm), 2,300 m, 21 Jan. 1968, *J.B. Gillett 18525* (BR***)**; Narok County: 17 miles from Olokurto on road to Elburgon, ±9,700 ft, 14 May 1961, *Glover, Gwynne & Samuel 1069* (K); Ol’Pusimoru sawmill about 9 miles from Olokurto, ± 8,500 ft, 20 May 1961, *Glover, Gwynne & Samuel 1336* (K***)**; Trans-Nzoia County: Mt. Elgon, Kenya, Saito Dam, 2,900 m, 3 Oct. 1997, *K. Wesche 1868* (K***)**; Suam Sawmills road, 1°9’N 34°45’E, 2,200 m, 21 May 1969, *D.M. Napper & Tweedie 2155.A* (K***)**; Mt. Elgon, Eastern slope above Tweedie’s saw-mill, 2,700 m s.m., 22 Feb. 1948, *O*. *Hedberg 117a* (S***,** UPS); Samburu County: Lolokwe (Donyo Sabachi), 00:52N 37:34E, 1,650 m, 15 Apr. 1977, *M.G. Gilbert 5369* (UPS, K***)**; Turkana County: Murna nysigar Peak, Lodwar Area, 25 Sep. 1963, *S. Paulo 1035* (BR**,** K***)**; Uasin Gishu County: Lake Narasha, 2,680 m, 0º3’N 35º32’E, 7 Sep. 1969, *J.L. Richardson 34* (K, MO**)**; Lake Narasha, 9,000 ft, at Equator in Timberoa, at lake margin, 17 Apr. 1966, *R. Wheeler Haires 128* (K); Lake Narasha, Timberoa, Kenya, 8,900 ft, 9 Feb. 1971, *D.A. Livingstone, T. Harvey, J. Melack & M. LaBarbera 71-9* (K); Western Province. Kakamega County: Eldoret-Kitale, halfway from Moi’s Bridge (Hoey’s Bridge) to junction with Bungoma road, 0º44’N 35º09’E, 8 Oct. 1981, *M. G. Gilbert & Mesfin Tadessa 6530* (ETH**)**; Nyanza Province. Nairobi, 19 Feb. 1947, *A. Bogdan 346* (K); Nairobi City, 6,000 ft, 30 Jan. 1966, *R. Wheeler Haires 4260* (K); The Carnivore (former Golf Driving Range), 1º18’S 36º48’E, ca. 1,700 m, 28 Jun. 1987, *R.B. Faden & F. Ng’weno 87/69* (UPS***)**; LESOTHO. Leribe [Hlotse], Basutoland, s.d., *A. Dieterlen 921* (PRE); Leribe [Hlotse], Basutoland, 1-2,000’, s.d., *A. Dieterlen 767* (SAM); Sehlabathebe Nat’l Park, Maal Cof., around 2,325 m, 3 Jan. 1979, *F.K. Hoener 2117* (S); Sehlabathebe National Park, 2,300 m, 5 Jan. 1990, *C. Schwabe #129* (MO); Sehlabathebe Nat’l Park, Matsa a Mafikeng, *A.C. Beverly & F.K. Hoener 522* (MO); Sehlabathebe Reserve, 9,100 ft, *R.D.A. Bayliss BS Lesotho 814* (MO**)**; Thaba Khupa on dam, ca. 1,600 m, 15 Nov. 1981, *M. Schmitz 9292* (MO); MALAWI. Dedza-Mphunzi Road, common plant along the Linthipe River bank, 22 Oct. 1978, *A.J. Salubeni 2361* (MO***)**; Mzimba Dist., Mzuzu, Marymount dambo, 4,500 ft, 13 Oct. 1973, *J. Pawek 7385* (LG, MO***)** & 20 Nov. 1973, *J. Pawek 7510* (MO**)**; Nyika National Park, Chelinda along rivulet near Chelinda chalet, 10°34’S 33°49’E, 2,300 m, 9 Dec. 1981, *L. van der Linden 250* (BR***)**; Nyika National Park, near *Juniperus* forest, 10°44’S 33°54’E, 13 Dec. 1981, *L. van der Linden 330* (BR***)**; N. Prov., Rumphid Dist., Nyika Plateau, Chelinda, edge of pond at Dam 3, 4 Jan. 1971, *J. Pawek 12262* (LG**)**; Zomba, s.d., *A. Whyte s.n.* (E, SAM); NAMIBIA. Otjozondjupa Region: Otjiwarongo Distr. [Otjiwarongo Constituency], Klein Waterberg, at ‘Schneiders Quelle’, 4 Apr. 1968, *L.E. Kers 2987* (S); Otjivarango District [Otjiwarongo Constituency], road Waterberg–Otjivarango [Otjiwarongo], farm Okosongomingo 149, 55 km SE of Otjiv., by ”Schneiders quelle” at Klein Waterberg, 4 Apr. 1968, *H. & HE. Wanntorp 660* (S); South West, Otjiwarongo, Waterberg Plateau Park, 22 Mar. 1984, *L. Smook 5197* (MO); SOUTH AFRICA. Höhe Hotteholland, s.d., *coll. unknown* *s*.*n*. (S); Free State Province. Witteberg, Molmonsspruit, 1875-1880, *A. Rehmann 3927* (WU); Lejweleputswa District Municipality: Hoopstad distr., Bultfontein, Oppermansdrifdam, 1,220 m, 15 Dec. 1974, *T.G. O’Connor 104* (MO); Gauteng Province. City of Johannesburg Metropolitan Municipality: Florida, Intersection of Hendrick Potgietrer Driveand Magongo Rd., 1,550 m, 13 Nov. 1984, *E.R. Robinson, J. Munday & D.W. Haines 13/11/84/016* (B**)** & *13/11/84/017* (B, E***)**; Melville Koppies Nature Reserve, Johannesburg, 22 Nov. 62, *[M Mocnae] 1507* (MO***)**; City of Tshwane Metropolitan Municipality: Bronkhorstspruit distr., 9 km east of roboy at Pick & Pay on Lynnwood Road, vlei source of Pienaars River, 1 Oct. 1980, *C. Reid 415* (BR***,** MO); bank of Krokodilspruit, 20 M N.E of Pretoria, 3,500’, 27 Mar. 1975, *P.C.V. du Toit s.n.* (MO**)**; ca. 20 km E of Pretoria along Witbank Freeway Donkelspoort, 20 Jul. 1982, *I. Kukkonen & M. Lassig s.n.* (H**)**; Water Kloof [Waterkloof], 12 Nov. 1919, *I.C. Verdoorn 5 I.C.* (BR); Sedibeng District Municipality: pr. Heidelberg, 1,660 m, 21 Oct. 1893, *R. Schlechter 3529* (WU); Helbron dist., Vereeniging: 5.5 mi. SSW. of Vereeniging, 1,435 m, 28 Nov. 1959, *J.P.H. Acocks 20974* (MO***)**; Xhariep District Municipality: Orange Free State, dam at Zastron campsite, 26 Mar. 1980, *C. Reid 151* (MO); KwaZulu-Natal Province. Drakensberg Garden, partie amont de la rivière Umzimkulu, réserve Garden Castle, env. 1,800 m, 8 Feb 1982, *J.* *Lambinon & M. Reekmans 82/421* (BR, LG); Mpendhle distr., Mulangane ridge, above Carter’s Nek. 7,000-7,300 ft, 9 Feb. 1989, *O.M. Hilliard & B.L. Burtt 17555* (E, S); Mpendhle distr., Mulangane ridge, above Carter’s Nek. 7,000-7,300 ft, 4 Feb. 1984, *O.M. Hilliard & B.L. Burtt 17557* (MO, S); Mpendhle distr., Mulangane ridge, above Carter’s Nek. 7,000-7,300 ft, 13 Mar. 1985, *O.M. Hilliard & B.L. Burtt 18374* (B, E); in humidis in Umzinyati River, Natal, Sep., *J.M. Wood 1053* (SAM); Amajuba District Municipality: pr. Newcastle, 1,300 m, 5 Oct. 1893, *R. Schlechter 3427* (WU); Sisonke District Municipality: Underberg District: 5-7 miles NNW of Castle View Farm, headwaters Mlahlangubo river, 7,000 ft, 21 Jan. 1982, *O.M. Hilliard & B.L. Burtt 15286* (E); Cobham Forest Reserve, Sipongweni, 6,500 ft, 22 Feb. 1981, *O.M. Hilliard & B.L. Burtt 14103* (E***)**; Garden Castle Forest Reserve, jeep track to Crystal Waters 6,000-6,200 ft, 5 Dec. 1980, *O.M. Hilliard & B.L. Burtt 13810* (E***)**; Garden Castle Forest Reserve, in marsh by stream near Forester’s house, ca. 6,000 ft, 4 Dec. 1980, *O.M. Hilliard & B.L. Burtt 137391* (E); vicinity of Tarn cave, above Bushman’s Nek, 8,000 ft, 19 Jan. 1984, *O.M. Hilliard & B.L. Burtt 17362* (E***,** S**)**; vicinity of Tarn cave, above Bushman’s Nek, 8,000 ft, 20 Jan. 1984, *O.M. Hilliard & B.L. Burtt 17429* (E); Ugu District Municipality: Port Shepstone distr., 14 miles on Port Shepstone–Izotsha Road, in ditch alongside the road, 15 Feb. 1968, *M. Ram 39* (E); Umzinto District, Vernon Crookes Nature Reserve, 400-500 m, 2 Feb. 1983, *K. Balkwill & J. Manning 1007 with T.F. Brophy & F.M. Getliffe Norris* (E); Umgungundlovu District Municipality: Mooi River, 10 Jan. 1901, *H.H. Johnston s.n.* (E); Mooi River, 4,000 ft, 30 Dec. 1899, *Mason s*.*n.* (SAM); Pieter-Mauritz-burg [Pietermaritzburg], Drakenberg, Oct. 1841, *J. Wahlberg s.n.* (S); Zululand District Municipality: Vryheid, Latemanek, Zommershoek farm, 1,814 m, 10 Apr. 1987, *B.J. Turner 1671* (MO); Limpopo Province. (Tzaneen), Wolkberg 634LT, 24 Apr. 1971, *P.J. Muller & J.C. Scheepere 184* (LISU); next to road on S. side of Kransberg mountain range, 16 May 1978, *A.A. Mauve 5051* (MO***)**; Mopani District Municipality: Letaba, Duiwelskloof [Modjadjiskloof], eastern bank of pool on Ramadiepa River, immediately below falls beneath Railway Bridge and Merensky Dam, 29 Sep. 1960, *J.C. Scheepers 1005* (B, S***)**; Sekhukhune District Municipality: near Maloeksekop, small swamp next to road (west side) from Bronkhorstspruit to Groblersdal, 18 Oct. 1977, *A.A. Mauve & F. Venter 5023* (MO); Waterberg District Municipality: mellan Warmbath och Nylstroom [Modimolle], 2 Oct. 1938, *A. Hafström & J.P.H. Acock 174* (B, S); Naboomspruit District, Stepping Stones Farm, owned by Mr. Rodger, 26 miles north-west of Naboomspruit [Mookgophong], Waterberg Mountain, Matthews Nature Reserve, 10 Oct. [19]72, *A.O.D. Mogg 37340* (MO); Mpumalanga Province. Ehlanzeni District Municipality: Lydenburg, De Kuylen, 2,050 m, 19 Feb., *G.G. Lavranos 15283* (E); Bei der Stadt Lydenburg, Dec. 1894, *F. Wilms 1564* (WRSL); Gert Sibande District Municipality: Bethal, 14 Dec. 1910, *R. Leendertz 3569* (K***)**; Wakkerstroom, distr. Ashock, ca. 2,100 m, 13 Dec. 1975, *N.J. Levenish 1612* (E***)**; Nkangala District Municipality: Elandskloof between Vleiland and Seweweekspoort, 4,700’, 21 Feb. 1986, *Moffett & Steensma 3886* (MO); Western Cape Province. Cape bona spei, s.d., *J. Wahlberg s.n.* (S); Cape Divisine about the ponds [...] at Salt River, s.d., *coll. unknown* *s*.*n.* (K**)**; Southern Cape, Outeniqua Mountains, on rd. between R62 and Prince Alfred’s Pass, 13 Dec. 1981, *P.B. Phillipson 526* (MO***)**; City of Cape Town: Brackenfel [Brackenfell], Nov. 1933, *J.P.H. Acock 2298* (S***)**; Stellenbosch Div., Somerset West, Oct. 1953, *R.N. Parker 4919* (SAM); Cape Winelands District Municipality: Regio occidentalis, in montibus pone Bainskloof, 1,500’, 7 Feb. 1897, *R.* *Schlechter 10259* (BR, E, L, MO, S***,** WRSL); Worcester beim Waterfall, Nov., *Drège* *s*.*n.* (E); Eden District Municipality: Hay, Witsand, ½ m W of Doornaar homestead, 4,100’, 25 Mar. 1959, *O.A. Leistner 1376* (B); pr. Riversdale, ± 300’, 3 Dec. 1892, *Schlechter 1938* (WU***)**; Overberg District Municipality: Goda, Hottentottsholland [Hottentots-Holland], Oct. 1841, *J. Wahlberg s.n.* (S***)**; Hottentottsholland [Hottentots-Holland], 14 Jan. 1875, *Gueinzius s*.*n.* (S**)**; Tradouw Pass, 500 m, 14 Jan. 1893, *R. Schlechter* *2084* (FR, WU); RWANDA. [...]–[...] Shangugu [Cyangugu], 2,000 m, 21 Mar.1956, *R. Christiaensen 1672* (LG*); Lac Bulera Riv., Gikongoro, Forêt de Nyungwe, environs de Muwasenkoko, km 67, 2,320 m, 17 Feb. 1971, *G. Bouxin 307* (MO); Forêt de Nyungwe, marais Muwasenkoko, 2,320 m, 11 Mar. 1971, *G. Bouxin 398* (MO); Kuwasenkoko, Berge marécageuse de la route rivière Senkoko, au pont de la route Astrida [Butare]-Bukavu, 2,350 m, 8 Feb. 1958, *J.-J. Symoens 5424* (BR); Nyamugania préfecture, Ruhenger, 29º48’E 1º30’S, 1,850 m, 15 Feb. 1972, *P. Bamps 3152* (LG**,** WAG); Rutovu Territorie dʹAstrida, préfecture Butare, 2,200, 02 Oct. 1958, G. Michel 5729 (LG*); Rutoru Territorie d’Astuda Ruanda, Forêt de Montagne, 2,200 m, 26 Oct. 1958, *G. Mitchel 5729* (BR); Nyamushia fourragères, Mar. 1929, *Scaetta 1716* (K); SOUTH SUDAN. Bushbuck Hill, 2,300 m, 14 Apr. 1982, *I. Friis & K. Vollesen 1208* (K***)**; between Gilo and Mt. Komoro, 4º03’N 32º53’E, 1,850 m, 18 Nov. 1980, *I. Friis & K. Vollesen 311* (K***)**; TANZANIA. Arusha Region: Ngorongoro Conserv. area: Mt Meru, Kilôto, 6 Oct. 1977, *J. Raynal 19482* (B, MO); Iringa Region: Dabanga Highlands, Kilolo, 24 ml. S.E. of Iringa, 6,100 ft, 10 Feb. 1962, *R. Polhill & S. Paulo* *1425A* (B); Mufindi District, Ngwazi, 8°31’S 35°10’E, 1,830 m, 6 Mar. 1989, *C.J. Kayombo & M.J. Kayombo 18* (MO); Stromgebiet des oberen Ruhudje, Landschaft Lupembe, nördlich des Flusses, Msima (Tsowi), 1931, *H.J. Schlieben 1034* (BR, S), Stromgebiet des oberen Ruhudje, Landschaft Lupembe, nördlich des Flusses, Apr. 1931, *H.J. Schlieben 648* (B) & *648a* (S); Stromgebiet des obern Ruhudje, Landschaft Lupembe, nördlich des Flusses, Rûhûdje, 18 Sep. 1931, *H.J. Schlieben 1211* (BR); Manyara Region: Basoda Lake, Mbulu, approx. 5,000 ft, Oct. 1925, *A.E. Haarer 13.A.* (K); Mbeya Region: Mbeya, 15 May 1957, *H.M. Richards 9730* (K***)**; Top of Chumala, […], edge of a stream [...] a Kita Kala, 5 Dec. 1963, *H.M. Richards 18573* (K); Poroto Mts., 2,340 m, 15 May 1957, *H.M. Richards 9730* (B); Rukwa Region: Malonje, Ufipa, 7,500 ft, 1949−1951, *A.A. Bullock 1890* (B***)**; Ufipa District, Mbisi [Mbizi] Forest, 2,550 m, 9 Nov. 1956, *H.M. Richards 6948* (B***)**; Ufipa, near Namwele, 4,500’, 10 Nov. 1963, *L.D.E.F. Vesey-FitzGerald 4222* (B); Ruvuma Region: Songea District, Matengo Hills, by R. Halau about 3 km. SE. of Miyau, 1,500 m, 12 Jan. 1956, *E. Milne-Redhead & P. Taylor 8314* (B***)**; Tanga Region: Leboma, Mar. 1893, *C. Holst 2556* (K, WRSL); Lushoto District, Mtai-Mlalo road, near Kidologwai, west Usambaras, 1,800 m, 19 May 1953, *R.B. Drummond & J.H. Hemsley 2644* (B, S); Usambaras, Oaklands, 5,200 ft, 17 Mar. 1970, *M. Batty 985* (K); UGANDA. Bog on Gahinga-Muhavura saddle, 1º23’S 29º39’E, 3,050 m, 24 Apr. 1970, *K.A. Lye & A. Katende 5290* (K); ZAMBIA. Serenje, petit marais en pente légère rive gauche de la Bolelo, 1,370 m, 23 Dec. 1963, *J.-J. Symoens 10675* (BR); ZIMBABWE. Manicaland Province. Harare: Kaola Estate, 7 mls. south of Salisbury [Harare], 14 Oct. 1955, *R.B. Drummond 4898* (B, S); Salisbury [Harare], 5,000 ft, Nov. 1919, *F. Eyles 1891* (SAM); Salisbury [Harare], University College of Rhodesia and Nyasaland site, 29 Mar. 1963, *J.P. Loveridge 640* (MO); Chipinga District: Kabanga Dam, Chipinga water supply dam 32°35’E 25°15’S, 26 Feb. 1973, *G.E. Gibbs Russell 2548* (MO); Makoni District: prope pagum Rusapi ad rivulum, ca. 1,400 m s.m., 26 Oct. 1930, *Th.C.E. Fries, T. Norlindh & H. Weimarck 2312* (BR***,** LISU, S); ca. 15 km. meridiem versus a pago Rusapi, ca. 1,450 m s.m., 10 Nov. 1930, *Th.C.E. Fries, T. Norlindh & H. Weimarck 2805* (S); Nyanga District: à 10 km à l’E.S.E. d’Inyanga [Nyanga] 18º16’S 32º49’E, 2,060 m, 30 Dec. 1973, *P. Bamps, J.J. Symoens & C. Vanden Berghen 445b* (BR); à 8 km au N.E. d’Inyanga [Nyanga], Lindi Waterfalls, 18º11’S 32º48’E, bord (rive gauche) de la rivière Lindi, 2,100 m, 31 Dec. 1973, *P. Bamps, J.J. Symoens & C. Vanden Berghen 470* (BR); Inyanga National Park [Nyanga National Park], Gaerezi R. drift, Gleneagles Rd., east of Mt. Inyagani, 5,200’, 3 Nov. 1973, *J.E. Burrows 125* (MO); Inyanga [Nyanga], ad villam Inyanga Down, ca. 1,900 m s.m., 29 Jan. 1931, *Th.C.E. Fries, T. Norlindh & H. Weimarck 4719* (BR, S***)**; Inyanga [Nyanga], ad pedem montis Inyangani, ad rivulum, ca. 1,900 m s.m., 5 Dec. 1930, *Th.C.E. Fries, T. Norlindh & H. Weimarck 3431* (BR, S); Inyanga [Nyanga] ad rivulum Niarerue, ca. 1,700 m s.m., 29 Oct. 1930, *Th.C.E. Fries, T. Norlindh & H. Weimarck 2408* (BR, S***)**; Inyanga [Nyanga], ad rivulum, ca. 1,700 m s.m., 19 Nov. 1930, *Th.C.E. Fries, T. Norlindh & H. Weimarck 3027* (BR, S***)**; Purdon Dam, 18º18’S 32º47’E, 1,951 m, 16 Aug. 1983, *P. Denny 1258* (MO); Mutare District: stream flowing out of Purdin Dam, Inyanga National Park [Nyanga National Park], 7 Jan. 1972, *G.E. Gibbs Russell 1240* (BR, MO); Mashonaland West Province. Suri Suri Dam, 16 Jun. 1972, *G.E. Gibbs Russell 1149* (MO).
