# Supplementary material for: Juncus quartinianus (Juncaceae, sect. Ozophyllum): A Neglected Species from the Horn of Africa and Its Re-Description Based on Morphological SEM Studies
Source: PLoS One. 2017 Jan 9;12(1):e0167838. doi: 10.1371/journal.pone.0167838 (PMC5221796; doi:10.1371/journal.pone.0167838)
Supplement: S2 Appendix — (DOCX) [file pone.0167838.s002.docx]

**S2Appendix. Specimens of *Juncus fontanesii* subsp. *pyramidatus* examined** (*specimens measured for statistical analyses).

Type : *Juncus pyramidatus* Laharpe, Essai Monogr. Jonc. 40 (1825); *Juncus fontanesii* var. *pyramidatus* (Laharpe) Buchenau, Bot. Jahrb. Syst. 1: 140 (1880). T: [label text by J. Gay: en Egypte par M. Savigny, donnée 1818] Egypt, M. J. C. Savigny (syn-: K 345807*).

*Juncus camptotropus* V.I. Krecz., Izv. Bot. Sada Akad. Nauk S.S.S.R 30: 98 (1932). T: [Turkey] Paphlagonia, wilajet Kastambuli, Tossia, ad ripas Devrek-tschai, 17 Jun. 1892, *P. Sintenis 4248* (iso-: B 100293621, B 100293622, BEI***,** BR–S.P. 688561, E 196513, JE 2602, K).

Representative specimens examined

CYPRUS. distr. Kyrenia: Boghazi, in ripa rivuli supra opp. Kyrenia, 9 Jun. 1939, *H. Lindberg s.n.* (H***)**; Episkopi, 11 May 1905, *J. Holmboe 706* (S***)**; distr. Paphos, monast. Ayios Neophytos, 21 Jul. 1939, *H. Lindberg* *s.n.* (H***)**; Mamonia, 8 May [19]66, [*M. Macdonald*] *161* (K*); near Prodromos, 5,000 ft, s.d., *A. Syngrassides 732* (K*); EGYPT. [Hamzma], 27 Apr. 1910, *coll. unknown s.n.* (K*); Abusir, Apr. 1906, *A. [Selerm] & Muschler s.n.* (BR), Aleksandria, Mar. 1902, *A. Arler s.n.* (K***)**, Bahariya Oasis: Western Desert, Al-Zabw, 3 May 1980, *M*. *Abd El Ghani 2646* (K**)**; Denderah [Dendera], Apr. 1906, *R. Muschler s.n.* (BR***)**; El Faiyum, 1973, *I. Mahdi, Sisi & A. Aziz s.n.* (H**)**; El Faiyum, 18 May 1975, *N. El Hadidi s.n.* (MO); El Fayûm N. f., Shakshûk wad, 26 May 1922, *N. D. Simpson 1300* (K); Fayoum: Sinnûrio [Sinnuris], 24 May 1986, *S. El Khanagry & B. Diwan 527* (K***)**; along the road from El Tell el Kebir to El Abbasia (El´ Abbâsa), ca. 2 km before El Abbasia (El´ Abbâsa), ca. 30º32’N 31º44’E, 14 May 1979, *C. van Vliet 357* (L, WAG); Itsa distr.: El-Mahmoudiya, 17 Apr. 1984, *M*. *Abd El Ghani 72/0* (K***)**; Near Tamia [Tamiya], 5 May 1967, *N. El Hadidi, M. Imam & K. Kosinova s.n.* (S***)**; Tamiya distr.: Kafr Mahfous [Mahfouz], 4 Jun. 1983, *M*. *Abd El Ghani 6519* (K***)**; Port Said, 27 Apr. 1910, *coll. unknown s.n.* (K); IRAQ. Baghdad, 30 Apr. 1958, *R. Wheeler Haires 201* (E***)**; 20 km E. of Dokan [Dukan], Suleimaniya Liwa [Sulaymaniyah], 27 May 1961, *coll. unknown s.n.* (MO***)**; Dorkor, Agrew (Haires), s.d., *R. Wheeler Haires s.n.* (E); Jarmo, nr. Chemchemal, 24 May 1955, *R. Wheeler Haires 201* (E***)**; Jarmo (Kirkuk), 24 May 1955, *R. Wheeler Haires W201* (K***)**; Jarmo, excavation Mound, 14 May 1955, *H. Helbaek 1757* (K***)**; Mindan Bridge, 270 m, 10 Jun. 1958, *E. Chapmar s.n.* (K***)**; Between Tell Afar and Balad Sinjar, 25 May 1934, *H. Field & Y. Lazar* *521* (S***)**; Zawita village, 750 m, 11 Jun. 1958, *E. Chapmar s.n.* (K**)**; ISRAEL–PALESTINE. Ain Meaharfah (Nakura) [An-Naqura], 2 m, 2 May 1923, *F.S. Meyers & J.E. Dinsmore B8825* (K***)**; Jaffa, May 1897, *J. Bornmüller 1565* (B***)**, Jerusalem, Ain Farah, 800 m, 20 May 1904, *J.E. Dinsmore 2825* (E); ebene Jesreël, Beth Alfa, -75 m, 13 Apr. 1936, *H. Bojko s.n.* (WU***)**; Latrun, 240 m, 26 Apr. 1905, *J.E. Dinsmore s.n.* (E); Tell el Kadi (Tall-ul-Quadi), 160 m, 11 May 1911, *F.S. Meyers & J.E. Dinsmore 8B.825* (E***,** H***,** S***)**; bei Sarona am Bachnfor, 22 May 1904, *A. Kneucker 358* (B***)**; banks of Wadi Rubin, 10 Jul. 1933, *A. Eig, N. Feinbrun & M. Zahary 220* (E, K***,** S***)**; JORDAN. Azraq Druze, by the spring Ain el’Enoquiry, 2,5 km N of the village, 30 May 1965, *C.C. Townsend 65/324* (K**)**; about midway Baq’aa-Jarash road, 17 Jun. 1974, *L. Boulos & W. Jallad 7271* (S); bed of Wadi Zerka between Suweileh & Jerash [Jarash], 5 Jul. 1941, *P.H. Davis 3776* (BR***,** E, K), Konawât [Gunnawat], Sep. 1875, *coll. unknown s.n.* (BEI**)**; Wadi Wala [Wadi al Wala], 21 Jun. 1975, *D. Al-Eisawi 2010*; LEBANON. Abeih [Aabey], Aug. 1864, *coll.* *unknown s.n.* (BEI***)**; near Beskinta, in muddy streamlet by roadside, 4,200 ft, 11 Jul. 1952, *H.F. Mooney 4545* (K***)**; Chouf, Mount Lebanon, Aïn Zhalta, 33º39’46”N 35º40’19”E, 1,193 m, 20 Jun. 2006, *J. Breidy & S. Khairallah LEB-524* (K***)**; Kesrouan, El Ghbaleh village, 34°3’4”N 35°43’44”E, 945 m, 30 Jul. 2004, *S. Khairallah & M. van Slageren MSSK 1618* (K); LIBYA: Djebel Nefousa [Nafusa Mountains], 1915, *Ch. d’Alleiztte s.n.* (L***)**; Kl. Oase: Quellsumpfen Anênah, 11 Apr. 1876, *P. Ascherson 519* (K, L***,** WRSL, WU***)**; SAUDI ARABIA. Asir: Al Habga near Baha, 20:00N 41:30E, 10 May 1992, *I. Hedberg & O. Hedberg 92136* (UPS***)**; waterfall, beauty spot between Abha v Jebel Sawdah, 8,500 ft, 16 Oct. 1981, *J.S. Collenette 2946* (K***)**; Bagasmar to Khadra, 27 Jul. 1976, *J.D. Dwyer 13762* (MO***)**; dirt road from Shumrukh to Seihan Province, 6,300 ft, 22 Apr. 1997, *J.S. Collenette 9481* (K***)**; Wadi Aalagah, 32 km S. of Baljurski Taif-Abha road, 6,000 ft, 29 Jul. 1982, *J.S. Collenette 3648* (K***)**; SYRIA. Hamath [Hama], Aug. 1883, *coll. unknown s.n.* (BEI***)**; Lattakia [Latakia], Jun. 1864, *coll. unknown s.n.* (BEI***)**, Marlian-Larytein [AL Qaryatayn], 20 Jul. 1890, *Nachel & Barbey* *s.n.* (BEI**)**; Ain Arus [Ain al-Arous] (fons fluvii Beleich) inter Rakka et Urfa, ad marginem locus Solola, ca. 350 m, 3 Jul. 1910, *H.F. v. Handel-Mazzetti 383* (WU**)**; TURKEY. Prov. Icel, Mersin ±3 km E of, ±2 m, 10 May 1959, *E. Hennipman, P. Nijhoff, C. Swennen, A.S. Tulp, W.J.M. Vader & W.J.J.O. de Wilde 1027* (B, L*, WAG***)**; YEMEN. In ditches Mahjur In Ahjir, ca. 2,800 m, 6 Oct. 1978, *J.R.I. Wood 2558* (K***)**; near Yarim, ca. 2,600 m, 16 Aug. 1977, *J.R.I.* *Wood* *1814* (K***)**; Yerin [Yarim], 17 Oct. 1986, *Acres 439* (K***)**; Y.A.R Distr., ca. 35 km N. of Taiz, around Dhisufal [Dhi as Sufal], ca. 1,800-2,100 m, 20 Oct. 1975, *F.N. Hepper & J.R.I. Wood 5896* (K***)**.
