# Supplementary material for: Juncus quartinianus (Juncaceae, sect. Ozophyllum): A Neglected Species from the Horn of Africa and Its Re-Description Based on Morphological SEM Studies
Source: PLoS One. 2017 Jan 9;12(1):e0167838. doi: 10.1371/journal.pone.0167838 (PMC5221796; doi:10.1371/journal.pone.0167838)
Supplement: S1 Table — (DOCX) [file pone.0167838.s003.docx]

| **Taxon** | **Country** | **Voucher** |
| --- | --- | --- |
| *Juncus oxycarpus* | South Africa | Tradouw Pass, 500 m, 14 Jan. 1893, *R. Schlechter* *2084* (FR) |
|  | South Sudan | Bushbuck Hill, 2,300 m, 14 Apr. 1982, *I. Friis & K. Vollesen 1208* (K) |
| *Juncus quartinianus* | Ethiopa | Amhara Region: ca. 115 km North of Lalibela along the road to Sekota, 12º33’N 39º04’E, 2,050 m, 27 Oct. 2001, *I. Fries, S. Bidgood, M. Wonderfrask & E. Getachew 10,804* (K) |
|  |  | Tigray Region: Adigerat (Adigrate) [Adigrat], 130 km S.S.E. of Asmara, 20 Jan. 1963, *T*.*H. Hages 170* (K) |
|  |  | South of Sinikala (Ferawen), 2,500 m, 17 Apr. 1992, *S*. *Demissew* *3191* (ETH) |
|  | Somalia | Sanaag: escarpment S of Laasqoray near Moon, 11:01N 48:25E, 1,300 m, 16 Jan. 1995, *M. Thulin, A. Dahir & A. Hassan 9072* (K) |
| *Juncus fontanesii* subsp. *pyramidatus* | Egypt | Western Desert, Al-Zabw, 3 May 1980, *M*. *Abd El Ghani 2646* (K) |
|  | Israel–Palestinae | Jerusalem, Ain Farah, 800 m, 20 May 1904, *J.E. Dinsmore 2825* (E) |
|  | Saudi Arabia | Waterfall, beauty spot between Abha v Jebel Sawdah, 8,500 ft, 16 Oct. 1981, *J.S. Collenette 2946* (K) |
|  |  | Wadi Aalagah, 32 km S. of Baljurski Taif-Abha road, 6,000 ft, 29 Jul. 1982, *J.S. Collenette 3648* (K) |
|  | Yemen | In ditches Mahjur In Ahjir, ca. 2,800 m, 6 Oct. 1978, *J.R.I. Wood 2558* (K) |

**S1 Table. Specimens used in the SEM examination of seeds**.
