# Supplementary material for: Juncus quartinianus (Juncaceae, sect. Ozophyllum): A Neglected Species from the Horn of Africa and Its Re-Description Based on Morphological SEM Studies
Source: PLoS One. 2017 Jan 9;12(1):e0167838. doi: 10.1371/journal.pone.0167838 (PMC5221796; doi:10.1371/journal.pone.0167838)
Supplement: S2 Table — (DOCX) [file pone.0167838.s004.docx]

**S2 Table. Results of Tukey’s HSD test for unequal sample sizes** (significant results (p<0.05) are given in bold).

| Character |  | *Juncus oxycarpus* | *Juncus quartinianus* | *Juncus fontanesii* subsp. *pyramidatus* |
| --- | --- | --- | --- | --- |
| Plant Hight | *Juncus oxycarpus* |  | **0.000105** | **0.000105** |
|  | *Juncus quartinianus* | **0.000105** |  | 0.212194 |
|  | *Juncus fontanesii* subsp. *pyramidatus* | **0.000105** | 0.212194 |  |
| Length of inflorescence | *Juncus oxycarpus* |  | **0.000076** | **0.000022** |
|  | *Juncus quartinianus* | **0.000076** |  | 0.659752 |
|  | *Juncus fontanesii* subsp. *pyramidatus* | **0.000022** | 0.659752 |  |
| Length of outer tepal | *Juncus oxycarpus* |  | 0.404281 | **0.000063** |
|  | *Juncus quartinianus* | 0.404281 |  | 0.168663 |
|  | *Juncus fontanesii* subsp. *pyramidatus* | **0.000063** | 0.168663 |  |
| Width of outer tepal scarious margin | *Juncus oxycarpus* |  | 0.997740 | **0.000022** |
|  | *Juncus quartinianus* | 0.997740 |  | **0.000080** |
|  | *Juncus fontanesii* subsp. *pyramidatus* | **0.000022** | **0.000080** |  |
| Length of inner tepal | *Juncus oxycarpus* |  | 0.963996 | **0.000199** |
|  | *Juncus quartinianus* | 0.963996 |  | **0.005305** |
|  | *Juncus fontanesii* subsp. *pyramidatus* | **0.000199** | **0.005305** |  |
| Width of inner tepal including scarious margin | *Juncus oxycarpus* |  | **0.011141** | 0.402373 |
|  | *Juncus quartinianus* | **0.011141** |  | 0.132643 |
|  | *Juncus fontanesii* subsp. *pyramidatus* | 0.402373 | 0.132643 |  |
| Width of inner tepal without scarious margin | *Juncus oxycarpus* |  | **0.000039** | 0.096312 |
|  | *Juncus quartinianus* | **0.000039** |  | **0.005823** |
|  | *Juncus fontanesii* subsp. *pyramidatus* | 0.096312 | **0.005823** |  |
| Length of anther | *Juncus oxycarpus* |  | **0.016782** | **0.000022** |
|  | *Juncus quartinianus* | **0.016782** |  | **0.000022** |
|  | *Juncus fontanesii* subsp. *pyramidatus* | **0.000022** | **0.000022** |  |
| Length of filament | *Juncus oxycarpus* |  | **0.000022** | **0.000022** |
|  | *Juncus quartinianus* | **0.000022** |  | **0.000022** |
|  | *Juncus fontanesii* subsp. *pyramidatus* | **0.000022** | **0.000022** |  |
| Anther/ filament length ratio | *Juncus oxycarpus* |  | **0.021236** | **0.000022** |
|  | *Juncus quartinianus* | **0.021236** |  | **0.000022** |
|  | *Juncus fontanesii* subsp. *pyramidatus* | **0.000022** | **0.000022** |  |
| Length of capsule | *Juncus oxycarpus* |  | **0.000022** | **0.000022** |
|  | *Juncus quartinianus* | **0.000022** |  | 0.450440 |
|  | *Juncus fontanesii* subsp. *pyramidatus* | **0.000022** | 0.450440 |  |
| Length of capsule mucro | *Juncus oxycarpus* |  | **0.000028** | **0.000022** |
|  | *Juncus quartinianus* | **0.000028** |  | **0.000022** |
|  | *Juncus fontanesii* subsp. *pyramidatus* | **0.000022** | **0.000022** |  |
| Width of capsule | *Juncus oxycarpus* |  | **0.000037** | **0.002103** |
|  | *Juncus quartinianus* | **0.000037** |  | **0.000022** |
|  | *Juncus fontanesii* subsp. *pyramidatus* | **0.002103** | **0.000022** |  |
| Capsule/perianth  ratio | *Juncus oxycarpus* |  | **0.000022** | **0.000022** |
|  | *Juncus quartinianus* | **0.000022** |  | **0.000022** |
|  | *Juncus fontanesii* subsp. *pyramidatus* | **0.000022** | **0.000022** |  |
| Number of heads in the inflorescence | *Juncus oxycarpus* |  | **0.013016** | **0.016459** |
|  | *Juncus quartinianus* | **0.013016** |  | **0.000026** |
|  | *Juncus fontanesii* subsp. *pyramidatus* | **0.016459** | **0.000026** |  |
| Number of flowers in the heads | *Juncus oxycarpus* |  | 0.354780 | **0.000022** |
|  | *Juncus quartinianus* | 0.354780 |  | **0.000035** |
|  | *Juncus fontanesii* subsp. *pyramidatus* | **0.000022** | **0.000035** |  |
